# Clinical value of wireless pH-monitoring of gastro-esophageal reflux in children before and after proton pump inhibitors

**DOI:** 10.1186/s12876-014-0225-7

**Published:** 2014-12-24

**Authors:** Michaela Boström, Ola Thorsson, Ervin Toth, Daniel Agardh

**Affiliations:** Department of Pediatrics, Unit of Endocrinology and Gastroenterology, Skåne University Hospital, Malmö, Sweden; Department of Clinical Physiology, Skåne University Hospital, Malmö, Sweden; Department of Gastroenterology, Endoscopy Unit, Skåne University Hospital, Malmö, Sweden

## Abstract

**Background:**

Wireless pH-monitoring is an accurate method for diagnosing adults with gastroesophageal reflux disease (GERD). The aim of this study was to evaluate the use of the Bravo capsule on children investigated for GERD in terms of safety, tolerability and feasibility before and after administration of proton pump inhibitors.

**Methods:**

A Bravo capsule was inserted during upper endoscopy under general anaesthesia or deep sedation with propofol. 48-hour pH-metry was performed in 106 children (50 males, 56 females) at the median age of 11 years (range 17 months-18 years). On the second day of investigation, proton pump inhibitor (PPI) was given at a mean dose of 1.6 mg/kg (SD ±0.6 mg). The definition of GERD was set to a reflux index (RI) of ≥5% and DeMeester score (DMS) ≥14.7.

**Results:**

Application of the capsule was successful in 103 of the 106 children (97.2%) and interpretable in 99 of these 103 (96.1%). 49 of the children with interpretable results (49.5%) had GERD according to RI, while 51 (56.7%) had GERD according to DMS. After PPI was given on day 2, RI decreased from a median of 4.9% (range 0.3-63.4%) to 2.2% (0–58.0%), while DMS decreased from a median of 17.6 (range 2.2-207.6) to 8.2 (0.3-178.6), respectively (p < 0.0001). No severe adverse events were reported.

**Conclusion:**

Wireless pH-metry is a safe and tolerable method when investigating children for GERD. PPI given on the second day of assessment provides additional information on response to treatment suggesting that pH-metry preferably should be extended to 48 hours.

## Background

Gastroesophageal reflux disease (GERD) is defined as a condition that develops when the reflux of stomach content into the esophagus causes troublesome symptoms and/or complications [1]. The most common GERD symptoms in adults are heartburn and regurgitation, with esophageal complications such as reflux esophagitis, hemorrhage, stricture, Barrett’s esophagus, and adenocarcinoma [1]. In children, GERD can also manifest as vomiting, poor weight gain, failure to thrive, dysphagia, cough, laryngitis, and wheezing [2,3]. It commonly occurs with regurgitation in healthy children from birth; however only 4% of the infants have daily signs of reflux at one year of age [4].

Ambulatory 24-hour esophageal pH-metry with a nasal wire system was previously considered the gold standard in diagnosing GERD [5]. However, a nasal catheter system is unpleasant for the patients and may restrict reflux-provoking activities, resulting in falsely lower values on pH-metry [6]. Therefore, a wireless system was developed using a capsule attached to the mucosa wall of the esophagus for electronic pH monitoring. This so-called Bravo pH Monitoring System (Medtronic Inc., Minneapolis, MN) has proven to be both safe and tolerable in adults [7,8], as well as among children [9,10]. The possibility of ambulatory pH-metry also facilitates diagnosing GERD [6]. In pediatric patients suspected to have GERD of whom many may be neurologically impaired or have certain genetic disorders [11], it is even more important to provide a less disturbing method for pH-metry.

Another advantage is the possibility of pH-monitoring for 48 hours [12]. By extending the measurement, the investigation will be more robust to intra-assay variations of refluxes over time. Still, there is no consensus whether a natural day to day variation of pH <4 should be considered when testing for reflux as some studies [13], but not others [14-17], demonstrate differences in refluxes over longer periods. By using 48-hour ambulatory pH-monitoring, the second 24 hours could alternatively be utilized to measure the effect of proton pump inhibitors (PPI).

To date, PPI are considered the most effective medications in inhibiting gastric acid secretion and have shown to decrease the number of reflux episodes in patients with GERD [18]. In adult patients screened for GERD, the wireless pH-monitoring system proved useful for evaluating the reduction of acid exposure during PPI medication given on the second day of measurement [19]. To the best of our knowledge, wireless pH-monitoring system testing with and without PPI has not yet been evaluated in children.

The aim of this study was to compile the results of the first 100 wireless pH-monitoring investigations performed on children screened for GERD at our pediatric gastroenterology unit in terms of feasibility, tolerability, and safety. A second aim was to evaluate if a second 24 hours of pH-metry gained further information to the diagnosis of GERD when the patient was given a high dose of PPI.

## Methods

A total of 106 children (50 males, 56 females) were consecutively screened for GERD with wireless pH-monitoring at a median age of 11 years (range 17 months-18 years). Patients were seen at the Department of Pediatrics between February 23^rd^, 2010 and March 3^rd^, 2013, in collaboration with the Unit of Endoscopy and the Department of Clinical physiology, at the Skåne University Hospital in Malmö, Sweden. All children were screened for clinical purposes of suspected GERD and all parents gave their informed consent to the investigation. Children on PPI medication were advised to discontinue treatment at least two weeks prior to the screening. The Skåne University Hospital research advisory board approved the review of medical records in accordance with the Declaration of Helsinki (SUS; 2013-04-22).

### Classification of GERD according to esophago-gastro-duodenoscopy (EGD) and histopathology

Under general anesthesia or deep sedation with propofol an EGD was performed and biopsies were taken from the distal parts of the esophagus. Minor signs of non-erosive esophagitis, such as shattered Z-line, increased vessel signs or thickened mucus membrane, were noted and the mucosa were also classified into grades according to the Los Angeles Classification regarding mucosal breaks [20,21]. A pathologist analyzed the biopsies and made a statement upon whether histological esophagitis existed according to the Ismail-Beigi scale [22].

### Application of the bravo capsule

During EGD, a Bravo pH Capsule (Medtronic Inc, Shoreview, MI, USA) was placed approximately 5 centimeters above the lower esophageal sphincter after being calibrated in buffer solution and activated by a magnetic switch, all according to manufacturer’s instructions. The capsule was attached to the esophageal mucosa by suction, and then secured in place by a stainless steel pin through the mucosa. The Bravo pH Capsule detects and transmits information of 6-second periods of pH-recordings every 12 seconds. A Bravo pH Receiver kept up to a maximum 2.5 meter from the patient recorded pH levels for approximately 48 hours. The patient and caregivers were instructed to record in a diary the position of the body (supine or upright), food intake, and symptoms throughout the 48-hour period. The second day of examination, the patients were instructed to be medicated with high dose PPI.

### Interpretation of pH recordings

The pH-measurements stored in the Bravo pH Recorder were analyzed using commercially available software (Polygram NET, SynMed Medical, Stockholm, Sweden). The 48 hours of data were displayed in a graph. Total number of reflux episodes (TRE), TRE under 5 minutes, time of the longest reflux episode (RE), and reflux index (RI) indicated by the percentage of time with pH <4, RI in an upright position, and RI in supine position were calculated. DeMeester score (DMS), a combination of the six different parameters mentioned above, was also calculated. This data, together with the symptoms recorded in the patient diary, were analysed for each the 24 hours during the 48-hour screening period. Data from the first 24 hours were used to classify the patient where limits of DMS ≥14.7 and RI ≥5% defined the presence of GERD.

### Statistical analysis

The data was analyzed in SPSS Statistics version 21 (SPSS, Inc., Chicago, IL). Asymmetric continuous data was presented with a median and range and analyzed with the Mann–Whitney U test and Wilcoxon signed rank test. A percentage of agreement, with kappa, was calculated on the outcome between EGD, histology and pH monitoring. Nominal data, as symptoms, were analyzed with McNemars test. A p-value <0.05 was considered statistically significant.

## Results

### Findings in EGD and histopathology

Of the 106 patients who underwent EGD, 61 (57.5%) had normal findings and 45 (42.5%) had macroscopic signs of esophagitis (Table 1). Biopsies from the esophagus were sent for histopathological evaluation in 104 of the 106 performed endoscopies (98.1%). Of these 104 children, 26 (25.0%) had esophagitis findings only in EGD, 9 (8.7%) had microscopical esophagitis only, 17 (16.3%) had esophagitis according to EGD that were confirmed by histology, and 52 (50%) children had normal findings with both examinations. The percentage of agreement between EGD and histology was 66.3% (κ = 0.26).

**Table 1:** **Outcome of upper endoscopy**

**Endoscopic finding**	**N (%)**
Minor signs of non-erosive esophagitis*	31 (30.7)
Erosive esophagitis classified by Los Angeles Classification:	
A	9 (8.9)
B	4 (4.0)
C	2 (2.0)
D	1 (1.0)
Columnar lined esophagus	6 (5.9)
Signs of hiatus hernia	1 (1.0)
Cardia insufficiency	1 (1.0)
Eosinofilic esophagitis	1 (1.0)
Normal	45 (45.6)

*Footnote*: Grading was performed according to the Los Angeles.

Classification. *Minor signs of non-erosive esophagitis were e.g. shattered Z-line, increased vessel signs or thickened mucus membrane.

### Attachment of the bravo capsule

In 3 of the 106 cases, capsules were not successfully attached for pH-metry. The first capsule was detached during the procedure, another capsule was inhibited due to failure of the Bravo pH Receiver prior to the investigation, and in the final case the capsule was not attached due to severe esophagitis. Application of the Bravo capsule was successful in the remaining 103 cases (97.2%) and attached at a mean of 4.8 centimeters above the lower esophageal sphincter (mean length of esophagus 34.1 cm).

### Feasibility of pH-monitoring

A complete pH-monitoring was successful in 99 of the 103 (96.1%) children with successfully attached capsules. There was one pH-measurement missing due to an error in the data transfer. Data on pH-monitoring were further excluded from three children; one child had a misplacement of the capsule, the capsule was detached in the second child, and the third child was continuing PPI during the whole procedure. Due to database error DMS was lost in 9 of the children with a complete pH-monitoring.

### pH-monitoring before PPI - Day 1

Of the 99 successful pH-monitoring measurements, the median of RI was 4.9% (range 0.3-63.4) and 49 (49.5%) children had RI ≥5%. The median of DMS was 17.6 (range 2.2-207.6) and 51 (56.7%) of the 90 children with available data on DMS had a DMS ≥14.7. Of the 90 patients with data on DMS the percentage of agreement of the patients with GERD according to RI and DMS was 90% (κ = 0.8). For the children with GERD according to DMS, the median RI was 10.7% (range 3.7-63.4), whereas the median DMS was 41.0 (range 17.9-207.6) among the children with GERD according to RI. The percentage of agreement between RI and EGD was 57.6% (κ = 0.15), between DMS and EGD was 51.1% (κ = 0.04), between RI and histology was 52.1% (κ = 0.03) and between DMS and histology 48.3% (κ = 0.04), respectively.

Diaries on meal intake, symptoms and body positions were completed by 96 of the 99 children with successful pH monitoring. From the 96 diaries, 87 children experienced one or more symptoms (Table 2) whereas 33 of the 90 children and 44 of the 99 children with successful pH monitoring, recorded one or more symptoms without having GERD according to DMS and RI, respectively.

**Table 2:** **Most common symptoms from diary records before (day 1) and after proton pump inhibitor (day 2) from 99 children**

**Symptom**	**Day 1**	**Day 2**	**p-value**
	**N (%)**	**N (%)**	
Stomach pain	31 (31.3)	32 (32.3)	1.00
Nausea	20 (20.2)	21 (21.2)	1.00
Chest pain	22 (22.2)	14 (14.1)	0.22
Reflux	20 (20.2)	10 (10.1)	0.40*
Vomiting	24 (24.2)	12 (12.1)	0.10*
Sore throat	15 (15.2)	7 (7.1)	0.18
Dysphagia	13 (13.1)	5 (5.1)	0.23
Burping	11 (11.1)	4 (4.0)	0.13
Water brash/Heartburn	8 (8.1)	4 (4.0)	0.22
Coughing	6 (6.1)	2 (2.0)	0.38

*after Bonferroni correction.

### pH-monitoring after PPI - Day 2

**Figure 1:**
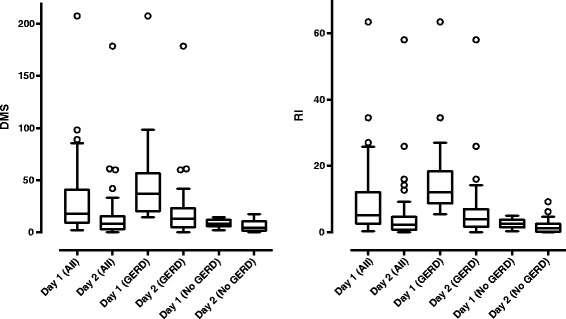
**Box plot of DeMeester score (DMS) and reflux index (RI) on day 1 and day 2 in all patients, and in patients with and without gastroesophageal reflux disease (GERD).** GERD was defined as having a DMS ≥14.7 and a RI ≥5, respectively.

Data on doses of PPI were available in 96 of the 99 children with successful pH-monitoring and was at a mean of 1.6 mg/kg (SD ±0.6). Data was missing in one case, and two of the children were treated with substances other than PPI and were therefore excluded from the analysis. After PPI was given on day 2, the median RI was 2.2 (range 0–58) and DMS was median 8.2 (0.3-178.6). There were a strong reduction of RI and DMS, 4.7% and 15.4 respectively (p < 0.0001; [95% CI 2.9-6.5, 9.3-21.5], respectively). Data on RI and DMS on days 1 and 2 are shown in Figure 1. Of the 96 children with data on PPI dosage, 94 also completed the diary on day 2 (Table 2). Of the 77 children who reported one or more symptoms, 57 of 96 and 46 of 87 of these children with available DMS did not have GERD according to RI and DMS, respectively.

### Tolerability and complications

Diaries were completed in 100 of 103 of the children who underwent wireless pH-monitoring. In one patient it was unclear if the recorded symptoms referred to the first or the second day. This patient was excluded from summoned symptoms (Table 2). Many patients recorded more than one symptom, but no child experienced severe symptoms that required intervention during the procedure. One child vomited one hour after the capsule placement, therefore no measurable values were registered. The same patient later vomited a small amount of blood. No other complication was recorded.

## Discussion

Through this retrospective study on children screened for GERD using the wireless Bravo Capsule system at a single pediatric unit, one can conclude that it is a safe and tolerable method of pH-monitoring in children. No severe adverse events was seen and no examination was terminated due to experienced symptoms, which is in line with previous studies performed on children [9,10]. The feasibility of the wireless pH-monitoring method was 96%, which is comparable to other studies reporting 97% and 86% successfully completed examinations [7,12].

The concordance between EGD and histology showed a scarce agreement whereas these two methods were even more discordant as compared with the wireless pH-monitoring. A plausible explanation for the discrepancy may be a biased evaluation by the pediatric gastroenterologist performing the endoscopy leading to an over-interpretation of esophagitis. Another reason for the discordant results could be the classification of esophagitis applied by the pathologist. Recent papers show that using the Ismail-Beigi scale is insufficient to detect microscopic esophagitis in some adult patients with non-erosive reflux disease (NERD) [23,24]. It is thus possible that the agreement between histology and endoscopy may have been improved if the biopsies had been reassessed according to the most recent guidelines.

Still, one third of the study cohort had no signs of esophagitis in EGD or microscopically, but had a pathological RI on pH-metry and would be misdiagnosed if not having been examined with pH-metry. Owing to the three methods included, children investigated in this study therefore seemed to benefit from pH-metry that caught the diagnosis in the majority of the patients including those having NERD. On the other hand, using the wireless Bravo Capsule system for pH-metry only records acid reflux and it cannot exclude that non-acid reflux is causing some symptoms. When using the multichannel intraluminal impedance and pH (MII-pH) monitoring system both acid and non-acid refluxes are analyzed, which enables to reveal any sort of reflux related to symptoms [25]. However, the disadvantage with the MII-pH method is the use of catheter which may be an unpleasant experience for the patient with risk for disturbing the investigation, especially when it is performed on children [6].

In this study, the upper limit of normal RI and DMS were set to 5% and 14.7, respectively. However, to date there are no standardized cutoff limits for wireless pH-monitoring for RI and DMS in adults or in children. Previous applied limits for pH-monitoring with conventional probes at 4.2% [26] for RI and 14.7 for DMS [27] correspond to a RI of 5.3% in adults with the wireless system [12]. For children, the likelihood of GERD is considered to be strong if RI is >7% and probable between a RI of 3-7% [28], whereas normal values for DMS in children has not been established. The cutoff limit set to 5% in this study was arbitrarily estimated. Therefore, further studies on children are needed to define normal values and optimal cutoff values for pathology in wireless pH-monitoring.

Today, many children are in general blindly treated with a short period of PPI prior to screening with EGD and pH-metry. A previous study clearly demonstrated that a PPI test has a low predictive diagnostic value for GERD [29]. The pH-monitoring in this study showed that 50% of the patients exceeded RI of 5% and 57% exceeded DMS of 14.7, so that they could successfully be treated for GERD. At the same time, approximately half of the children in our study cohort did not have GERD. They probably would have been treated with PPI with potential risks for adverse effects if they had not been screened properly [30]. In addition, significant costs due to long periods of unnecessary PPI treatments can be avoided [31].

The effect of PPI has previously been documented in children and adolescents examined with MII-pH monitoring [32]. In that study, measures before and after a two-month medication period revealed significant reduction in RI and DMS as well as symptom improvement despite the number of total reflux being unchanged. The number of acid reflux decreased when the number of non-acid or weakly acid reflux increased. By giving a high dose PPI on day 2 in our study, we were also able to demonstrate a strong reduction of both RI and DMS after PPI was given to the children that were diagnosed with GERD. This corresponds with another study of adult patients on and off PPI during pH metry [19]. The procedure with and without high dosage of PPI also enables the investigator to select those individuals that do not respond to ordinary doses of PPI and would instead benefit from surgery.

On the other hand, there are some limitations for not utilizing the measurement of crude pH on both days. Due to the day-to-day variability in acid exposure there is a small risk of missing some children for not being screened for 48 hours under normal conditions [13]. Adult patients that differ in acid exposure from one day to another seem to have a lower total esophageal acid exposure than the patients that do not show variation in reflux over time [33]. Another potential pitfall is the use of anesthesia or deep sedation with propofol when performing the endoscopy. It cannot be disregarded that the use of sedation may have an effect on the lower esophagus sphincter function immediately after the procedure resulting in increased numbers of acid refluxes on pH-metry the first hours of examination. There is thus a potential risk that this subgroup of children could have been misdiagnosed with GERD in the present study. However, other parameters such as clinical signs and GERD related symptoms in relation to body position and meal intake were also studied in correlation to the pH recordings that could also provide valuable information to the diagnosis.

Interestingly, there was no apparent overall difference in reported symptoms between children diagnosed with and without GERD. No specific symptom was reduced on the second day of investigation as a response to PPI. Among the children without GERD according to pH-metry, three of four children still suffered from some sort of symptom during the first day of the examination. The most common recorded symptoms in this study were chest and stomach pain. Chest pain and foreign body sensation due to the capsule have previously been described [8,9,12]. Since we did not collect information on the children’s symptoms prior to the study, no conclusion could be drawn of whether some symptoms where caused by the capsule. Yet, no patient experienced such severe pain that the screening had to be interrupted by surgical or endoscopical intervention. More importantly, no severe adverse effects were reported of the method in our cohort. However, the risk of anesthesia and performing endoscopy with biopsies cannot be disregarded [34]. It is therefore recommended to reassure the safety of the method through frequent evaluations.

## Conclusions

This study shows that the Bravo Capsule system for esophageal pH-monitoring is both safe and tolerable in children. The overall feasibility was 96%, which is considered high from a single unit as compared to other studies. By extending the pH-metry to 48 hours, the method offers the possibility to study the dose effect of PPI on acidic refluxes and effect on self-reported symptoms in patients diagnosed with GERD. We therefore recommend that in cases where pH-metry is needed, the Bravo Capsule wireless system is used as the standard screening method of suspected GERD in children.
